# Determinants of cerebral blood flow and arterial transit time in healthy older adults

**DOI:** 10.18632/aging.206112

**Published:** 2024-09-18

**Authors:** Jack Feron, Katrien Segaert, Foyzul Rahman, Sindre H. Fosstveit, Kelsey E. Joyce, Ahmed Gilani, Hilde Lohne-Seiler, Sveinung Berntsen, Karen J Mullinger, Samuel J. E. Lucas

**Affiliations:** 1 School of Sport, Exercise and Rehabilitation Sciences, University of Birmingham, Birmingham, UK; 2 Centre for Human Brain Health, University of Birmingham, Birmingham, UK; 3 School of Psychology, University of Birmingham, Birmingham, UK; 4 College of Psychology, Birmingham City University, Birmingham, UK; 5 Department of Sport Science and Physical Education, University of Agder, Kristiansand, Norway; 6 Institute of Inflammation and Ageing, University of Birmingham, Birmingham, UK; 7 Sir Peter Mansfield Imaging Centre, School of Physics and Astronomy, University of Nottingham, Nottingham, UK

**Keywords:** ageing, arterial transit time, cardiorespiratory fitness, cerebral blood flow, cognitive function

## Abstract

Cerebral blood flow (CBF) and arterial transit time (ATT), markers of brain vascular health, worsen with age. The primary aim of this cross-sectional study was to identify modifiable determinants of CBF and ATT in healthy older adults (*n* = 78, aged 60–81 years). Associations between cardiorespiratory fitness and CBF or ATT were of particular interest because the impact of cardiorespiratory fitness is not clear within existing literature. Secondly, this study assessed whether CBF or ATT relate to cognitive function in older adults. Multiple post-labelling delay pseudo-continuous arterial spin labelling estimated resting CBF and ATT in grey matter. Results from multiple linear regressions found higher BMI was associated with lower global CBF (β = −0.35, *P* = 0.008) and a longer global ATT (β = 0.30, *P* = 0.017), global ATT lengthened with increasing age (β = 0.43, *P* = 0.004), and higher cardiorespiratory fitness was associated with longer ATT in parietal (β = 0.44, *P* = 0.004) and occipital (β = 0.45, *P* = 0.003) regions. Global or regional CBF or ATT were not associated with processing speed, working memory, or attention. In conclusion, preventing excessive weight gain may help attenuate age-related declines in brain vascular health. ATT may be more sensitive to age-related decline than CBF, and therefore useful for early detection and management of cerebrovascular impairment. Finally, cardiorespiratory fitness appears to have little effect on CBF but may induce longer ATT in specific regions.

## INTRODUCTION

Brain health worsens with age [1] and ultimately impairs cognitive function, limiting independence in later life [2]. These effects will impact many as the global population ages rapidly [3]. Alongside adverse age-related structural and functional cerebral deterioration, changes to cerebral haemodynamics occur, including widespread cerebral hypoperfusion [4]. Evidence indicates that lower cerebral blood flow (CBF) detrimentally affects cognitive function in healthy older adults [5–8]. Identifying strategies that limit adverse age-related changes to cerebral haemodynamics could help promote a healthier ageing process; however, modifiable determinants of cerebral haemodynamics in older adults are currently poorly understood.

Not only CBF but arterial transit time (ATT) also worsens with age [4]. ATT is the time taken for blood to travel from large arteries in the neck to the cerebral tissue. Prolonged ATT is associated with impaired cerebrovascular reactivity [9] and atherosclerotic risk [10], and is present in patients with Alzheimer's disease [11] or cerebral artery stenosis [12]. The MRI sequence arterial spin labelling (ASL) can estimate both CBF and ATT if data are acquired at multiple post-labelling delays. Using multiple post-labelling delays also improves CBF estimation accuracy by enabling adjustment for regional and individual differences in ATT [13]. Despite this, compared with single-delay ASL, only a minority of ASL studies have utilised this technique due to increased data collection time requirements, although shorter multi-delay sequences are now available [14].

Previous research in older adults has already identified some modifiable determinants of CBF. For example, greater CBF is reported in those who are physically active [15, 16], engage with social or leisure activities [17], have a lower body mass index (BMI) [18, 19], or have lower blood pressure [20, 21]. These factors can all be addressed with simple lifestyle changes. Cardiorespiratory fitness is a modifiable factor for which its relationship with CBF is unclear, despite evidence that it benefits cognitive function [22, 23] and reduces dementia risk [24]. Research in older adults has reported a positive relationship between cardiorespiratory fitness and CBF [25–28], and that exercise training can increase CBF [28–32]. In contrast, a negative [33, 34] or a lack of [35–37] association has also been reported. In summary, when observed, cardiorespiratory fitness-related CBF changes are not usually global, but confined to specific regions of the brain [31–33, 38]. Regions are often small or only a portion of regions investigated show these associations [27, 30–32, 34]. Furthermore, none of the aforementioned ASL studies reporting effects used multiple post-labelling delays, limiting accuracy of CBF estimation [39]. The large genetic component of cardiorespiratory fitness could also explain discrepancies in results [40]. Given the complexity and inconsistency in the literature to date, more work is needed to understand the relationship between these variables using improved methodological approaches.

Research investigating modifiable determinants of ATT is limited, but evidence indicates a positive association with mean arterial pressure [21] and an unexpected negative association with BMI in males with coronary artery disease (limited to two small regions within the brain) [41]. A proxy of ATT, the spatial coefficient of variation in ASL signal (sCoV) [42], appears to lack association with hyperlipidaemia [43]. Regarding cardiorespiratory fitness, one study investigated relationships with ATT, reporting a lack of or positive relationship in older (*n =* 14) or younger (*n =* 18) adults, respectively [35]. Blood velocity within a cerebral artery is also somewhat of a proxy for ATT because of a strong inverse relationship [35], and also demonstrates mixed associations with cardiorespiratory fitness [44]. Originally, a positive association between cerebral blood velocity and cardiorespiratory fitness was shown in males [45] whereas more recent work indicates that this association is not present [46] or potentially only present in females [47]. Given that ATT has been related to brain health outcomes [9, 11, 12], further research is required to understand its determinants.

This cross-sectional study aimed to investigate determinants of global and regional resting CBF and ATT in healthy older adults, with a particular focus on cardiorespiratory fitness, and whether CBF or ATT are associated with cognitive function. Resting grey matter CBF and ATT were estimated using pseudo-continuous ASL with multiple post-labelling delays. It was hypothesised that markers of superior general health (i.e., higher cardiorespiratory fitness/handgrip strength/grey matter volume or lower age/BMI/blood pressure) and cognitive function would be associated with greater CBF and a shorter ATT.

## RESULTS

Mean global and regional values for CBF and ATT can be found in Supplementary Table 1 and Supplementary Figure 1.

### Determinants of global CBF

**Figure 1:**
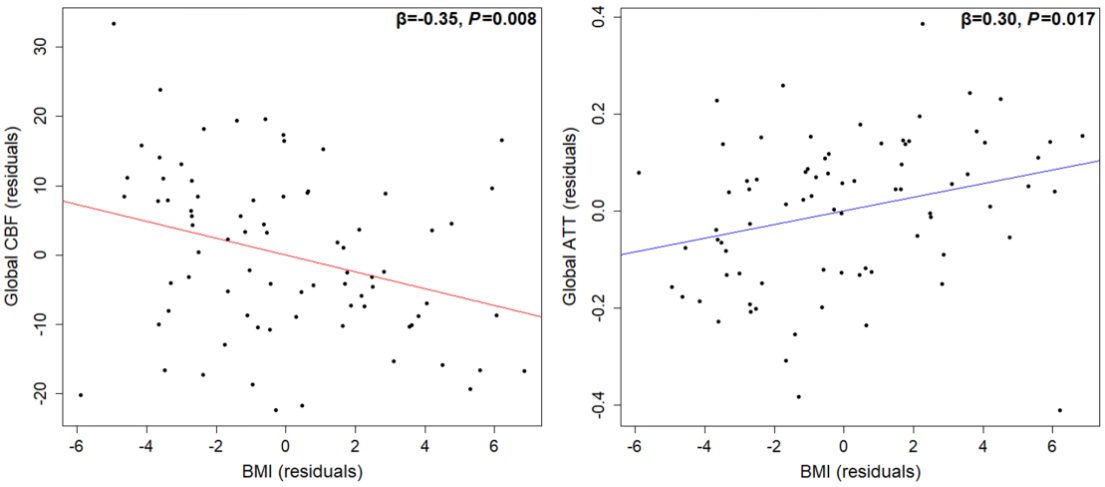
**Partial regression association between BMI and global CBF (left) or global ATT (right), adjusted for age, sex, blood pressure, cardiorespiratory fitness, hand grip strength, and grey matter volume.** Higher BMI was associated with lower CBF and a longer ATT (*n* = 78). Abbreviations: CBF: cerebral blood flow; ATT: arterial transit time; BMI: body mass index.

Overall, the eight independent variables did not significantly explain the variance in global CBF (gCBF) (F (8, 69) = 1.38, *P* = 0.22, R^2^_adjusted_ = 0.38). BMI was the only significant determinant of gCBF (β = −0.35, *P* = 0.008; Figure 1), whereby gCBF decreased with increasing BMI. Data shown in Table 1.

**Table 1:** Determinants of global resting CBF and ATT in grey matter.

	**gCBF (mL/100 g/min)**		**gATT (s)**	
***n* = 78**	**β**	***P***	**β**	***P***
Age (years)	−0.16	0.298	0.43	**0.004**
Sex (1; male, 2; female)	0.03	0.895	−0.19	0.406
BMI (kg/m^2^)	−0.35	**0.008**	0.30	**0.017**
SBP (mmHg)	−0.02	0.877	−0.11	0.371
DBP (mmHg)	−0.11	0.429	0.06	0.651
V̇O_2peak_ (mL/kg/min)	−0.18	0.304	0.24	0.149
Hand grip strength (kgf)	0.06	0.775	−0.33	0.115
Grey matter volume (mm^3^)	0.06	0.687	−0.01	0.943

Results from two multiple linear regression analyses. Bold indicates *P* < 0.05. Abbreviations: β: standardised beta coefficient; gCBF: global cerebral blood flow; gATT: global arterial transit time; SBP: systolic blood pressure; DBP: diastolic blood pressure; BMI: body mass index; V̇O_2peak_: peak oxygen consumption.

### Determinants of global ATT

**Figure 2:**
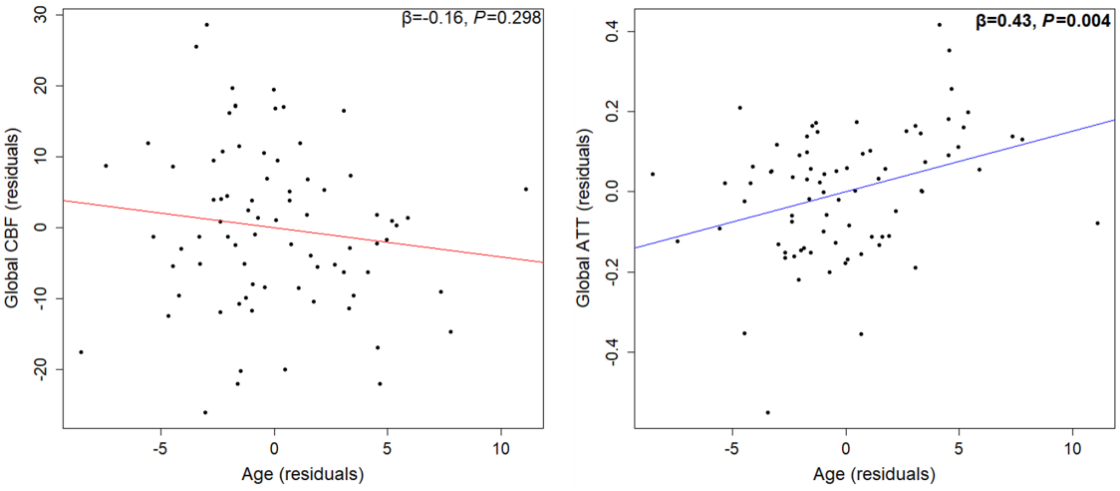
**Partial regression association between age and global CBF (left) or global ATT (right), adjusted for sex, BMI, blood pressure, cardiorespiratory fitness, hand grip strength, and grey matter volume.** ATT may be more sensitive to age-related changes than CBF (*n* = 78). Abbreviations: CBF: cerebral blood flow; ATT: arterial transit time; BMI: body mass index.

Overall, the eight independent variables did significantly explain the variance in global ATT (gATT) (F (8, 69) = 2.66, *P* = 0.013, R^2^_adjusted_ = 0.15). Only age (β = 0.43, *P* = 0.004; Figure 2) and BMI (β = 0.30, *P* = 0.017; Figure 1) were the significant determinants of gATT, whereby gATT lengthened with both BMI and age. Data shown in Table 1.

### Associations between regional CBF and ATT with age, BMI, and cardiorespiratory fitness

### Regional CBF

Age and cardiorespiratory fitness were not significantly associated with CBF of any region, before or after adjustment for multiple comparisons. Negative associations between BMI and CBF were present in all regions after adjustment for multiple comparisons, with the largest associations in the temporal (β = −0.44, *P* < 0.001), occipital (β = −0.43, *P* < 0.001), and parietal (β = −0.41, *P* = 0.002) regions. Data were shown in Supplementary Table 2.

### Regional ATT

**Figure 3:**
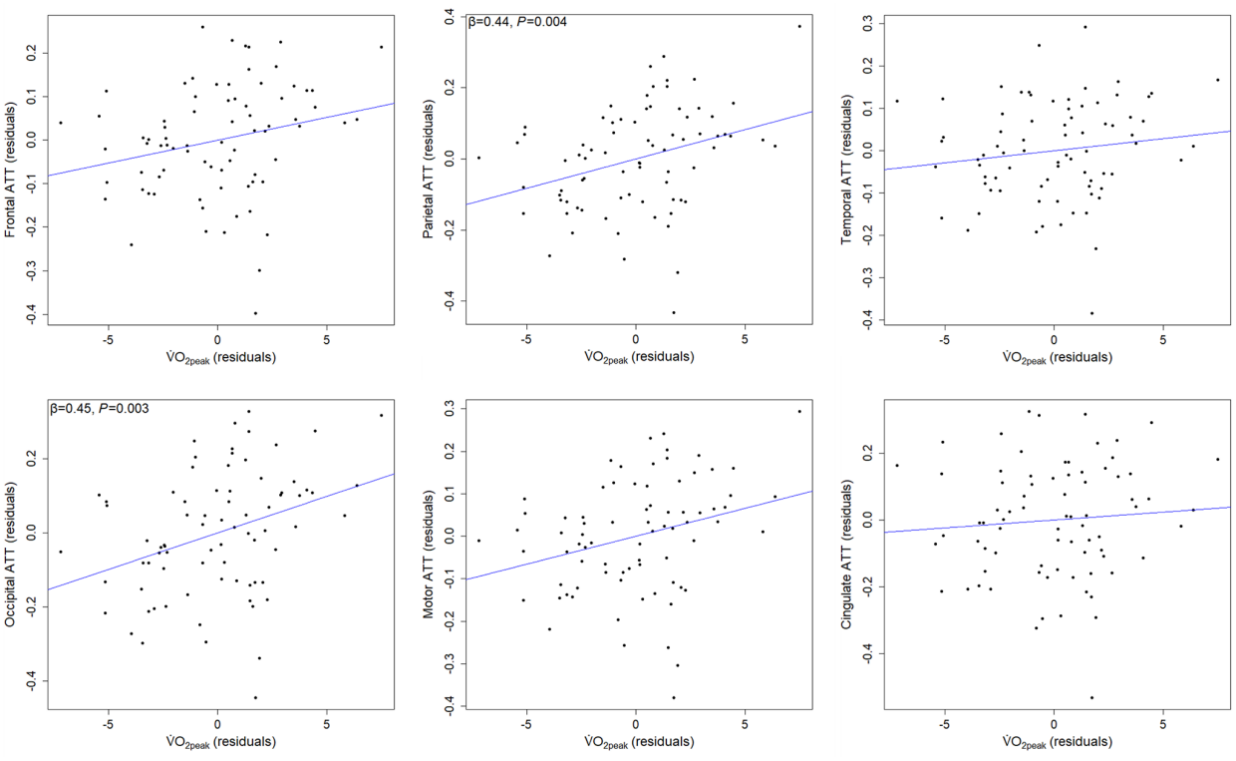
**Partial regression associations between regional ATT and cardiorespiratory fitness, adjusted for age, sex, and BMI.** Older adults with higher cardiorespiratory fitness experience longer ATT in parietal and occipital regions (*n* = 77). Abbreviations: ATT: arterial transit time; BMI: body mass index; V̇O_2peak_: peak oxygen consumption.

Full regional ATT results are shown in Table 2. Age was positively associated with ATT in all regions (strongest in occipital), only the cingulate region did not survive adjustment for multiple comparisons. Significant positive associations between BMI and ATT were present in frontal, parietal, temporal, and motor regions, but these did not survive adjustment for multiple comparisons. Cardiorespiratory fitness was positively associated with ATT in frontal, parietal, occipital and motor regions, and associations in parietal (β = 0.44, *P* = 0.004) and occipital (β = 0.45, *P* = 0.003) regions survived adjustment for multiple comparisons (Figure 3).

**Table 2:** Associations between age, BMI, and cardiorespiratory fitness with regional ATT.

**ATT**	**Age**		**BMI**		**V̇O_2peak_**	
	**β**	***P***	**β**	***P***	**β**	***P***
Frontal	0.47	**<0.001**	0.24	0.046	0.33	0.042
Parietal	0.57	**<0.001**	0.23	0.040	0.44	**0.004**
Temporal	0.46	**<0.001**	0.25	0.043	0.20	0.225
Occipital	0.62	**<0.001**	0.19	0.083	0.45	**0.003**
Motor	0.49	**<0.001**	0.26	0.034	0.40	0.012
Cingulate	0.32	0.017	0.24	0.063	0.12	0.495

Separate multiple linear regressions were performed for each region (*n* = 77), independent variables: age, sex, BMI, and V̇O_2peak_. Bold indicates significant *P*-values survived adjustment for multiple comparisons. Abbreviations: β: standardised beta coefficient; ATT: arterial transit time; BMI: body mass index; V̇O_2peak_: peak oxygen consumption.

### Associations between cognitive function and global CBF or ATT

Overall, the models including all independent variables did not significantly explain variance in gCBF (R^2^_adjusted_ = −0.017, F (9, 66) = 0.86, *P* = 0.56) or gATT (R^2^_adjusted_ = 0.031, F (9, 66) = 1.27, *P* = 0.27). Measures of processing speed, working memory, or attention were not significantly associated with gCBF or gATT. The only noteworthy result between both models was that processing speed accuracy approached significance for predicting gCBF (β = 0.25, *P* = 0.053). Global regression data and summarised results from regional analyses can be found in Supplementary Table 3 and section Associations between cognitive function and regional CBF or ATT of the Supplementary Materials, respectively.

## DISCUSSION

The present study aimed to identify modifiable determinants of CBF and ATT in healthy older adults and assess whether CBF and ATT are associated with cognitive function. The use of multiple post-labelling delay ASL improved CBF estimation accuracy by adjusting for differences in ATT, which is especially important in older populations [13]. The present data show older adults with a higher BMI had lower global CBF and a longer global ATT, and that global ATT lengthened with age. Sex, blood pressure, cardiorespiratory fitness, hand grip strength, and grey matter volume were not significant determinants of global CBF or ATT. Regional analysis confirmed a lack of association between cardiorespiratory fitness and CBF but indicated that older adults with a higher cardiorespiratory fitness experience longer ATT in parietal and occipital regions. Cognitive function was not associated with CBF or ATT, globally or regionally.

### Higher BMI is associated with lower CBF and prolonged ATT in healthy older adults

BMI was the only significant determinant of global CBF, whereby higher BMI was associated with lower CBF (1.2 mL/100 g/min decrease per 1 kg/m^2^ increase). This finding agrees with previous population-based research [19, 48]. A novel finding from the present study was that higher BMI was also associated with prolonged global ATT. This opposes previous research reporting a negative association in male coronary artery disease patients (limited to two clusters within the occipital lobe) [41]. Making a direct comparison with the present data is problematic due to differences in the populations studied. The present findings reinforce the known damaging effects of excessive weight gain on brain vascular health.

Mechanisms mediating the relationship between BMI and poor cerebrovascular health may relate to the prevalence of metabolic and vascular risk factors that increase with BMI (e.g., hypertension, arterial stiffness, or hyperlipidaemia) [49–51]. These risk factors worsen cerebrovascular health [52, 53] and are associated with lower CBF [54–56]. Given that only 17% of the present sample were taking lipid-lowering medication and that blood pressure or grey matter volume were not significant determinants of global CBF or ATT, it is unlikely that higher blood pressure, hyperlipidaemia, or cerebral atrophy typically associated with higher BMI [50, 57] explain the observed results. Therefore, adverse structural and functional changes to peripheral and cerebral vessels associated with higher BMI [49, 51] are potentially the mediators of these associations.

Taken together, poor metabolic health and excessive weight gain have deleterious effects on brain vascular health. Fortunately, research indicates that modifying dietary and physical activity habits to induce ~10 kg weight loss increases CBF in overweight and obese middle-aged adults [58]. Evidence also indicates that engagement with high, but not low or moderate, levels of physical activity ameliorate the CBF reductions observed with higher BMI [19]. The impact of weight loss interventions on ATT warrants further investigation.

### Cardiorespiratory fitness lacks association with CBF, whereas associations with ATT may be region-specific

There are conflicting findings regarding the association between cardiorespiratory fitness and CBF in older adults with only one previous ASL study using multiple post-labelling delays [35]. The present data found no association between cardiorespiratory fitness and global or regional CBF. Other cross-sectional ASL research also reports no global effect [35, 37, 38], but positive regional associations are generally observed [25–28, 38]. However, these associations relate to smaller regions than those investigated in the present study, and previous studies used single-delay ASL to estimate CBF. Furthermore, cardiorespiratory fitness has a large genetic component [40] and refers primarily to the efficiency of oxygen delivery/utilisation at skeletal muscle, not the brain, potentially explaining the present findings.

Cardiorespiratory fitness was not a significant determinant of global ATT, but higher cardiorespiratory fitness was associated with longer ATT in parietal and occipital regions, opposing our hypothesis. The only other study investigating this association in older adults (*n =* 14) reported no global association but regional analysis, investigating the same regions as the present study, did show non-significant prolongation of ATT in high-fitness older adults, likely due to sample size [35]. Collectively, the present data indicate that, in healthy older adults, cardiorespiratory fitness does not alter the delivery rate of perfusion of blood to cerebral tissue, but instead lengthens the time taken for blood to arrive at parietal and occipital regions from larger cerebral arteries in the neck.

The cardiorespiratory fitness-related prolongation of regional ATT could be related to blood velocity, vascular path length, or both. Faster cerebral artery blood velocity is associated with shorter ATT [35]; however, its relationship with cardiorespiratory fitness is unclear [44]. A positive association between blood velocity and fitness was first documented in males [45] but this has failed to be replicated [46] or has been replicated only in females [47]. This ambiguity, along with evidence that masters athletes experience less age-related increases in cerebral vessel tortuosity [59], suggests neither increased large artery blood velocity nor vascular path length explains the present findings. However, masters athletes also have more small cerebral vessels [59]. Therefore, given that total vessel cross-sectional area is inversely proportional to blood velocity (assuming constant blood flow), cardiorespiratory fitness-induced small vessel cerebral angiogenesis may be slowing blood velocity and thus prolonging regional ATT. Interestingly, a longer ATT or larger sCoV (its proxy) has been associated with greater oxygen extraction fraction in patients with cerebrovascular disease [60, 61], potentially indicating that overall slower cerebral blood velocities may translate into longer capillary transit times, thus improving oxygen extraction. Therefore, fitter older adults may have superior cerebral oxygen extraction, which is true in skeletal muscle [62], and could help explain the preservation of cerebral tissue integrity and cognition that is associated with regular exercise training.

### Age was not a significant determinant of CBF, but was associated with prolonged ATT

Age-related CBF decline over the lifespan is well documented [4, 20, 21], even after partial volume effects due to age-related grey matter atrophy are accounted for [63]. However, such findings were not replicated in the present data (with partial volume correction), even when using simple correlations (Supplementary Table 4), probably due to the limited age range. Previous research using a similar age range also reported no age/CBF relationship [64]. Yet, age was associated with prolonged global and regional ATT in the present study, conforming with previous findings [4]. The fact that both age and cardiorespiratory fitness are associated with a longer ATT in older adults is somewhat contradictive; however, the cause likely differs. Age-related ATT prolongation could be caused by adverse structural cerebrovasculature changes, such as increased cerebral vessel tortuosity [65, 66] and prevalence of cerebral stenosis [67], or by reductions in cerebral artery blood velocity [45, 46]. Despite this, age-related ATT prolongation may consequently serve to improve oxygen extraction fraction (similarly to cardiorespiratory fitness) and help explain why the cerebral metabolic rate of remaining cerebral tissue increases with age [68]. Given that age was a significant determinant of ATT but not CBF, ATT may be more sensitive to age-related decline and could therefore be used to identify the onset of cerebrovascular impairment in older adults with low cardiorespiratory fitness.

### Blood pressure was not a determinant of CBF or ATT

The present study found no association between global CBF or ATT and blood pressure in healthy older adults (systolic blood pressure = 140–160 mmHg in 49%). Alternative multiple linear regressions were performed with either mean arterial pressure or pulse pressure, but this did not affect results (data not shown). Previous single-delay research using ASL found higher systolic and diastolic blood pressure was associated with lower global CBF in older adults (34% hypertensive) [20]. Furthermore, multiple-delay research reports higher mean arterial pressure is associated with lower global CBF in all age groups and longer ATT only in a limited number of regions [21]. However, longitudinal changes in blood pressure and global CBF were not associated in hypertensive older adults [69]. Interestingly, hypertension is actually suggested to be a protective response to cerebral hypoperfusion in an attempt to maintain CBF [70]. The relationship between blood pressure and cerebral haemodynamics is clearly complex and thus between-study differences may be explained by variance in the severity or duration of blood pressure changes experienced. However, given that higher blood pressure increases the risk of cerebrovascular dysfunction [71] and dementia [72], it can be assumed that maintaining normal blood pressure is beneficial for brain health. Further multiple-delay longitudinal research is required to make robust conclusions regarding the short- and long-term effects of blood pressure on CBF and ATT.

### No association between CBF or ATT and cognitive function

Chronic cerebral hypoperfusion is thought to contribute to cognitive decline in older adults [73]. However, the present data found no association between CBF and processing speed, working memory, or attention in healthy older adults. This agrees with previous research showing global and regional CBF were not associated with cognitive function in older adults [74]. Interestingly, however, a subset of this previous sample was followed-up two years later which found lower baseline global CBF predicted greater decline in global cognition and attention/psychomotor speed whereas specifically frontal and temporal CBF predicted memory decline [6]. Collectively, these data do suggest the importance of CBF for cognitive function, but this is both domain- and region-specific, and only apparent when changes over time are considered. Regarding ATT, the present study is believed to be the first to investigate relationships with cognitive function, reporting no associations. Previous research has investigated relationships with clinical cognitive impairment, reporting that prolonged ATT or its proxy, sCoV, is present in participants with vascular cognitive impairment, vascular dementia, or Alzheimer’s disease [11, 43].

Taken together, it appears that CBF and ATT lack association with contemporaneous cognitive function in healthy older adults, but changes in these measures may still predict changes in cognitive function over time, as seen with CBF [5–7]. Given that the present data indicate greater age-related sensitivity of ATT than CBF, the predictive capacity of ATT should be investigated as it could indicate cerebrovascular-related cognitive decline in healthy populations. Alternatively, rather than resting state cerebral haemodynamics (i.e., oxygen delivery), oxygen utilisation of the cerebral tissue may be more important for cognitive function.

### Future directions

Cross-sectional analysis does not account for variations in genetics or baseline vascular health, and longitudinal/intervention research is needed to make robust conclusions. There are likely other influential determinants of cerebral haemodynamics not investigated in the present study, such as physical activity, arterial stiffness, cerebrovascular reactivity, blood lipids, or social activities that deserve future investigation. Regarding cognition, future research should investigate the predictive capacity of CBF and ATT. General brain health may be more strongly dictated by the ability of the cerebral tissue to extract and use essential nutrients delivered in the blood (i.e., oxygen extraction fraction and cerebral metabolic rate). Future research should investigate associations between these variables with cardiorespiratory fitness, CBF, ATT, and cognitive function. We used a range of PLDs based on the optimal for ATT <2000 ms [75]. This appears sufficient for our relatively healthy older population as shown by the observed ATTs (Supplementary Figure 1). However, the longest PLD used (2300 ms) may not be sufficient to capture more prolonged ATT in older adult samples that are older or less healthy and should be considered in future studies. The present study lacked acute dietary controls prior to MRI acquisition (e.g., caffeine, polyphenols, nitrate, or sugars), which could have impacted results [76–78]. Future studies should measure and correct for arterial partial pressure of CO_2_ (*P*aCO_2_), or its proxy, partial pressure of end-tidal CO_2_ (*P*etCO_2_) [79]. These were not measured during CBF/ATT measurements but could be manipulated by anxiety-induced ventilatory changes during an MRI scan. Accelerometer-derived activity measurements were conducted as part of the larger project, revealing 85% of participants met the activity guidelines they self-reported not to, based on moderate-to-vigorous intensity activity [80], although participants possibly modified their normal behaviours [81]. The generalisability of findings may therefore be limited, and future research should assess if differences in CBF and ATT exist between habitual high and low activity groups. Power analysis in the present study indicated power = 0.61 (pwr, RStudio). Given that previously documented associations were observed (e.g., age/ATT or BMI/CBF), power was sufficient to detect medium-to-strong effects. Larger sample sizes may be required to detect smaller effects and may explain why cardiorespiratory fitness/ATT associations were not present globally.

## CONCLUSIONS

This study aimed to identify modifiable determinants of CBF and ATT in healthy older adults and assess whether CBF and ATT were associated with cognitive function. Higher BMI was associated with lower global CBF and longer global ATT. Cardiorespiratory fitness was not associated with CBF, but fitter older adults unexpectedly had prolonged ATT in parietal and occipital regions. Blood pressure and grip strength were not associated with CBF or ATT. Interestingly, data indicate greater age-related sensitivity of ATT than CBF. Regarding cognitive function, neither CBF nor ATT were associated with contemporaneous processing speed, working memory, or attention. Future research should investigate responses of CBF and ATT to exercise training and whether CBF or ATT predict changes in cognitive function over time.

## MATERIALS AND METHODS

### Study design

The data for this publication were collected as part of a larger study, The FAB Project (preregistration: https://osf.io/6fqg7, materials and data: https://osf.io/d7aw2/). The study was approved by the STEM Ethical Review Committee at the University of Birmingham (ERN_20-1107).

**Figure 4:**
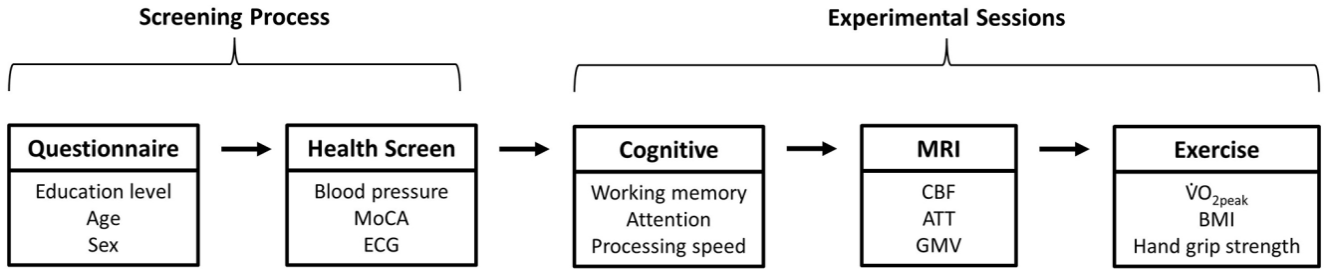
**Flow chart of the screening and experimental sessions.** Abbreviations: MoCA: Montreal Cognitive Assessment; ECG: electrocardiogram; MRI: magnetic resonance imaging; CBF: cerebral blood flow; ATT: arterial transit time; GMV: grey matter volume; V̇O_2peak_: peak oxygen consumption; BMI: body mass index.

Participants were screened for eligibility before completing three experimental sessions on different days. Figure 4 shows the key outcome measures and desired session order (achieved for 87% of participants, with all experimental sessions completed within 5.2 ± 3.2 weeks, and with 13 ± 15 days between MRI and exercise sessions (<30 days for 91%). Participants refrained from vigorous physical activity, which acutely alters CBF [76], for 24 hrs prior to the MRI session.

### Participants

Ninety-four healthy older adults (aged 60–81 years) were enrolled. Participants were cognitively normal, without historic or current diagnosis of serious health conditions, non-smokers, and self-reported to not meet recommended global activity guidelines [82]. The section Participant inclusion criteria of the Supplementary Materials contains detailed inclusion criteria. MRI data were missing or unusable for *n =* 16, leaving *n =* 78 for analyses presented in this study. Participant characteristics are shown in Table 3.

**Table 3:** Participant characteristics.

	**Total**	**Male**	**Female**
*n*	78	39	39
Age (years)	65 ± 5	65 ± 5	66 ± 5
Education (%)			
Compulsory	28	31	26
Further	33	33	33
Undergraduate	19	18	21
Postgraduate	19	18	21
SBP (mmHg)	140 ± 13	140 ± 12	139 ± 15
DBP (mmHg)	82 ± 8	83 ± 7	82 ± 8
BMI (kg/m^2^)	27 ± 4	28 ± 3	26 ± 4
V̇O_2peak_ (mL/kg/min)	28 ± 4	30 ± 4	25 ± 3

Values represent means ± standard deviation. Abbreviations: SBP: systolic blood pressure; DBP: diastolic blood pressure; BMI: body mass index; V̇O_2peak_: peak oxygen consumption.

### Health screening: electrocardiogram (ECG), blood pressure, and cognitive impairment

Participants completed a resting 12-lead ECG (Cardiosoft, Vyaire, USA), three resting blood measurements (705IT, Omron, Japan), and the Montreal Cognitive Assessment (MoCA). Participants were excluded for severe ECG abnormalities, MoCA scores <23 [83], and systolic/diastolic blood pressure of >160/>90 mmHg. Excluded participants were referred to their GP.

### Outcome measures

### Cardiorespiratory fitness

Participants completed an incremental exercise test on a treadmill (Pulsar 3p, H/P/Cosmos, Germany). Respiratory gases (V̇O_2_; oxygen consumption, V̇CO_2_; carbon dioxide production) were recorded continuously using a facemask (7450 V2, Hans Rudolph, USA) and metabolic cart (JAEGER Vyntus CPX, Vyaire, USA), as was heart rate and rhythm using a 12-lead ECG (Cardiosoft, Vyaire, USA). Rating of perceived exertion (RPE) [84] and finger-prick blood (lactate) (Biosen C-Line, EKF Diagnostics, United Kingdom) were measured between stages. Stages were 4 min with a 1 min rest period between each stage. Treadmill speed started and remained at 3.8 km/h until either all possible elevation stages were completed (4, 7, 10, 13, 16, 19, and 20% gradient) or individual lactate threshold was reached (2.1 mmol/L increase over the mean of the two lowest values [85]). If all elevation stages were completed, 4 min stages continued with speed increasing 0.5 km/h per stage until lactate threshold. After reaching lactate threshold, 1 min stages were completed where speed increased 0.5 km/h per stage (rest periods were removed). Supplementary Figure 2 shows a treadmill test format example.

Participants were asked to exercise to volitional exhaustion unless halted by the researcher due to ECG abnormalities or injury. Cardiorespiratory fitness was determined using peak oxygen consumption (V̇O_2peak_) (i.e., mean of the two highest 30 s intervals). Nine participants completed a sub-maximal test. V̇O_2peak_ was thus predicted using individual sub-maximal V̇O_2_ and heart rate data acquired from three of the first possible six stages using linear regression. Full details and example (Supplementary Figure 3) of the prediction method can be found in section Cardiorespiratory fitness test of the Supplementary Materials.

### Hand grip strength

Hand grip dynamometer (5001, Takei, Japan) was adjusted for grip size in the participant’s dominant hand. Participants stood upright, arms by the sides, and squeezed the dynamometer as hard as possible, maintaining elbow extension and limiting shoulder movement. The highest score from three attempts was taken.

### Cognitive function

*Working memory*

In a 2-back task, participants were presented a 3 × 3 grid. The stimulus was a single white square that continuously appears, disappears, and then reappears in one of the grid squares at random (*n =* 60 trials, 1 s each). Participants identified when the white square appeared in the same location as it did two trials prior. Trials were excluded from analysis if incorrect or if response time <200 ms or greater than two standard deviations above/below the mean per participant. The primary outcome measure was *d* prime (*d*’), a measure of discriminability, a greater *d*’ indicates superior performance.

*Attentional network task (ANT)*

The computerised ANT assessed orienting, alerting, and executive control. The stimulus is a row of five arrows, each pointing left or right. As fast and as accurately as possible, participants reported the direction of the centre arrow using the left and right arrow keys. A central fixation cross is displayed for 400 ms, then a fixation cross (500 ms) and cue (100 ms) are presented simultaneously, and then only the fixation cross is displayed for a further 400 ms. A stimulus is then shown for a maximum of 1700 ms. The centre arrow can be congruent or incongruent (i.e., pointing in the same or opposite direction as the flankers, respectively; *n =* 96 each), or neutral (i.e., central arrow flanked by target-irrelevant black blocks, *n =* 96). The stimulus can appear above or below the fixation cross, cued by a black square (*n =* 216) or not cued (*n =* 72). There are three cue conditions: a spatial cue, a centre cue, or a double cue (*n =* 72 each). The spatial cue indicates if the stimulus appears above or below the fixation cross, whereas the stimulus location remains ambiguous for the centre and the double cue. Twelve practice trials are followed by three blocks of 96 trials.

Trials were excluded from analysis if incorrect or if response time <200 ms or greater than two standard deviations above/below the mean per participant. Alerting scores were calculated as the no cue minus the double cue; Orienting scores by centre cue minus the spatial cue; Executive control scores were calculated by the incongruent target minus the congruent target (all for correct responses). High condition difference scores for alerting and orienting, and low condition difference scores for executive control, indicate better performance.

*Processing speed*

In a letter comparison task, participants were simultaneously presented with two strings of letters at the top and bottom of the screen, for a maximum of 2500 ms after presentation of a fixation cross (1000 ms). Strings were three or six characters long (*n =* 48 trials, 24 each). As fast and as accurately as possible, participants identified whether the strings were the same or different. Mean response time and accuracy were calculated for each participant using data from trials involving only six-character strings. Trials were excluded from analysis if incorrect or if response time <200 ms or greater than two standard deviations above/below the mean per participant.

### MRI data acquisition and analysis

An MRI scan session included structural, functional, and arterial spin labelling (ASL) scans, using a 3-T system (MAGNETOM Prisma, Siemens, Germany) with 32-channel receiver head coil. Here, the focus is the ASL data and related scans; analysis of other data acquired can be found elsewhere [86]. CBF and ATT data were collected using pseudo-continuous ASL scan with 3D GRASE readout (17:22 mins) [87, 88], see also Acknowledgements.

*ASL imaging parameters*

Repetition time (TR) = 4100 ms, echo time (TE) = 30.56 ms, in-plane resolution = 3.5 mm^2^, slice thickness = 3.5 mm, transversal slices = 32, field of view (FOV) = 224 × 224 mm, labelling duration = 1508.8 ms, background suppression = yes, and post-labelling delays (PLD) = 200, 975, 1425, 1850, 2025, 2150, 2250, and 2300 ms. Four and twelve volumes of data were acquired for PLD of 200–2250 ms and 2300 ms, respectively. PLD times and number of volumes acquired were optimised according to recommendations [75]. Slices were positioned axially from the motor cortex and angled anterior-posterior in line with the participant’s anterior-posterior commissure (ACPC). A calibration M_0_ scan was acquired using these same parameters with the PLD set to 2000 ms. The T1-weighted structural scan (4:54 mins) was acquired to facilitate data analysis including, normalisation to a standard template brain and differentiation of grey and white matter. Structural T1-weighted (MPRAGE) imaging parameters were: TE = 2.03 ms, TR = 2000 ms, voxel size = 1 mm^3^, sagittal slices = 208, FOV = 256 mm, and flip angle = 8°.

ASL data were processed using the Oxford ASL toolbox (https://oxasl.readthedocs.io/en/latest/), which uses the FSL FABBER ASL package and Bayesian Inference to invert the kinetic model for ASL MRI (BASIL) to compute CBF and ATT maps [89–91]. Parameters input to the kinetic models to estimate CBF and ATT were: bolus duration = 1.5088 s, tissue T1 = 1.3 s, arterial blood T1 = 1.65 s, and labelling efficiency = 0.85. All other input parameters were kept with default settings appropriate to PCASL acquisition. Partial volume error correction and adaptive spatial smoothing of the perfusion maps were performed using default settings in oxford_asl [90, 92].

Global and regional analysis was performed, assessed in native (individual participant) and MNI space, respectively. All CBF and ATT values refer to grey matter only. Regions of interest (ROI) were the cingulate gyrus and frontal, parietal, temporal, occipital, and motor cortices (Supplementary Figure 4). The chosen ROIs have been used previously [35], and were broad because there were no specific *a priori* hypotheses of regions that would be affected by determinants or associated with cognitive function. MNI registration was poor for *n =* 1, leaving *n* = 77 for regional analysis. Difference maps at each PLD for each participant were visually inspected to ensure data quality. Particular attention was paid to ensure there were no: (1) excessive motion resulting in spurious edge effects in difference maps; (2) brain territories which did not appear to be perfused, due to suboptimal label positioning or unaccounted-for vasculature; and (3) focal areas of high intensity in final CBF maps which would have suggested that the PLDs were insufficient. Examples of participants which were excluded after visual inspection are shown in Supplementary Figure 5. Section MRI acquisition and analysis of the Supplementary Materials contains additional information regarding grey matter mask configuration.

Grey matter volume was estimated from structural T1 anatomical scans. Brain extraction tool (BET) removed non-brain tissue [93] before segmentation of tissue types using FMRIB’s Automated Segmentation Tool (FAST) [94].

### Statistical analyses

All statistics used multiple linear regressions (SPSS Statistics v.29, IBM, USA). To identify determinants, global CBF or ATT were the dependent variable with age, sex, blood pressure (systolic and diastolic), BMI, V̇O_2peak_, hand grip strength, and grey matter volume as independent variables. To assess regional associations with cardiorespiratory fitness, mean CBF or ATT of each of the six ROIs were the dependent variable with age, sex, V̇O_2peak_, and any other significant determinants of global CBF or ATT identified from the above analysis as independent variables. To identify associations with cognitive function, global CBF or ATT were the dependent variable with age, sex, education, and scores for processing speed (accuracy and response time), working memory (d’), and the three attentional domains (alerting, orienting, and executive control scores) as independent variables. Cognitive data were missing for *n =* 2, leaving *n =* 76 for global analysis. The same analyses were performed using regional data, presented in section Associations between cognitive function and regional CBF or ATT of the Supplementary Materials.

## Supplementary Materials

Supplementary Material

Supplementary Figures

Supplementary Tables
