# Supplementary material for: Determinants of cerebral blood flow and arterial transit time in healthy older adults
Source: Aging (Albany NY). 2024 Sep 18;16(18):12473–97. doi: 10.18632/aging.206112 (PMC11466485; doi:10.18632/aging.206112)
Supplement: Supplementary Material [file aging-16-206112-s001.pdf]

SUPPLEMENTARY MATERIALS

METHODOLOGY

Participant inclusion criteria

Inclusion criteria were: (1) aged 60–85 years; (2) do <150 mins of moderate physical activity per week; (3) monolingual; (4) right-handed; (5) no current/historic diagnosis of cardiovascular, metabolic, respiratory, neurological, kidney, liver, or cancerous disease; (6) resting electrocardiogram (ECG) and blood pressure screened by clinician (i.e., no severe ECG abnormalities (e.g., ST depression, long QT, heart block, wide QRS) and systolic/diastolic blood pressure of <160/<90 mmHg, respectively); (7) Montreal Cognitive Assessment (MoCA) score ≥23; (8) not taking neurotransmitter-altering medication; (9) vaccinated against COVID-19; (9) deemed safe to enter MRI scanner by a qualified MRI operator (i.e., absence/very small amount of ferrous metal in the body); (10) no language impairments; (11) no post-traumatic stress disorder (PTSD); (12) non-smoker of at least 5 years.

Cardiorespiratory fitness test

Treadmill test format example

Oxygen consumption ($\dot{V}O_2$) and carbon dioxide production ($\dot{V}CO_2$) measured continuously. Lactate and RPE measured during each rest period and 1 min post-exercise. Heart rate recorded at the end of each stage. Participants began the next stage whilst waiting for lactate analysis from the previous rest period.

Peak oxygen consumption prediction method

Peak oxygen consumption ($\dot{V}O_{2peak}$) was predicted using the equation: x = (y-c)/m. Gradient (m) and intercept (c) were calculated from the line of best fit between three sub-maximal heart rate and $\dot{V}O_2$ data points from an individual's treadmill test, and y = age-predicted maximal heart rate ($HR_{age\text{-}pred}$; 220-age). This method assumes a largely linear relationship between heart rate and $\dot{V}O_2$ and that $HR_{age\text{-}pred}$ is relatively accurate (generally ± 8–12 bpm) [1]. This method was tested on 13 participants that completed high-quality treadmill tests (peak values: $\%HR_{age\text{-}pred}$ = 103 ± 5, respiratory exchange ratio (RER) = 1.14 ± 0.03). The mean difference between actual ($\dot{V}O_{2peak}$) and predicted ($\dot{V}O_{2peak\text{-}pred}$) peak oxygen consumption was 8.6 ± 2.5% (range = −11–12.3%). For n = 8, the difference was <10%.

The difference in the slope of the regression lines when using three and six sub-maximal stages is minimal. For this participant, predicted peak oxygen consumption ($\dot{V}O_{2peak\text{-}pred}$) was underestimated (−5.1%) when using $HR_{age\text{-}pred}$ (151 bpm; green line). For this participant, accuracy of this method was improved by 2% when using the peak heart rate (HR_{peak}) recorded in the treadmill test (160 bpm; red line). This method was validated in a sub-sample of participants who completed high-quality treadmill test (n = 13), mean change in predictive accuracy when using $HR_{age\text{-}pred}$ to HR_{peak} = 2.1 ± 3.4).

MRI acquisition and analysis

As described in the main manuscript, for each participant, ASL data at each PLD (difference of tag and control averaged over repeats of each PLD) and grey matter masks in native space. Participants with abnormal ASL data were further investigated and n = 6 data were excluded due to excessive motion. Supplementary Figure 5 shows some examples of ASL data which were excluded after this visual data quality inspection step. Native space grey matter masks were thresholded at 0.5 probability to ensure only voxels containing primarily grey matter were included in calculations of CBF and ATT. Areas within masks containing incorrect assignment to grey matter, primarily around the eyes and nasal cavity, were manually removed (n = 7).

Structural MRI data were aligned to the MNI brain using fsl_anat (https://fsl.fmrib.ox.ac.uk/fsl/fslwiki/fsl_anat). Registrations to MNI space were visually inspected. For participants with poor registration, the nonlinear registration was disabled and processing re-run. For participants whose registration was deemed poor despite best efforts (due to brain atrophy with age), data were excluded from regional analysis requiring data in MNI space (n = 1). Regional grey matter masks were made in MNI space and defined using the MNI structural atlas (temporal lobe only) or from the conjunction of the relevant regions from the Harvard atlas (in FSL). For n = 9, MNI registration specifically of the inferior frontal lobe was poor (due to significant brain atrophy) and thus frontal lobe CBF and ATT maps were edited to exclude this region from analysis using fslroi.

RESULTS

Associations between cognitive function and regional CBF or ATT

To analyse regional differences, multiple linear regressions were performed with regional CBF or ATT as dependent variables, and age, sex, education (1; compulsory, 2; further, 3; undergraduate, 4; post-graduate) and scores for processing speed (accuracy and

response time), working memory (*d* prime), and the three attentional domains (alerting, orienting, and executive control response times) as independent variables. MNI registration was poor for $n = 1$ and cognitive data were missing for $n = 2$, leaving $n = 75$ for regional analysis.

All associations between CBF or ATT of any region and any cognitive measures were non-significant. Only processing speed accuracy showed any indication of a trend with non-significant positive associations with CBF in all regions ($\beta = 0.23$–0.25, $P = 0.052$–0.080).

REFERENCES

1. Achten J, Jeukendrup AE. Heart rate monitoring: applications and limitations. Sports Med. 2003; 33:517–38. https://doi.org/10.2165/00007256-200333070-00004 PMID:12762827
