# Supplementary material for: Determinants of cerebral blood flow and arterial transit time in healthy older adults
Source: Aging (Albany NY). 2024 Sep 18;16(18):12473–97. doi: 10.18632/aging.206112 (PMC11466485; doi:10.18632/aging.206112)
Supplement: Supplementary Figures [file aging-16-206112-s002.pdf]

SUPPLEMENTARY FIGURES

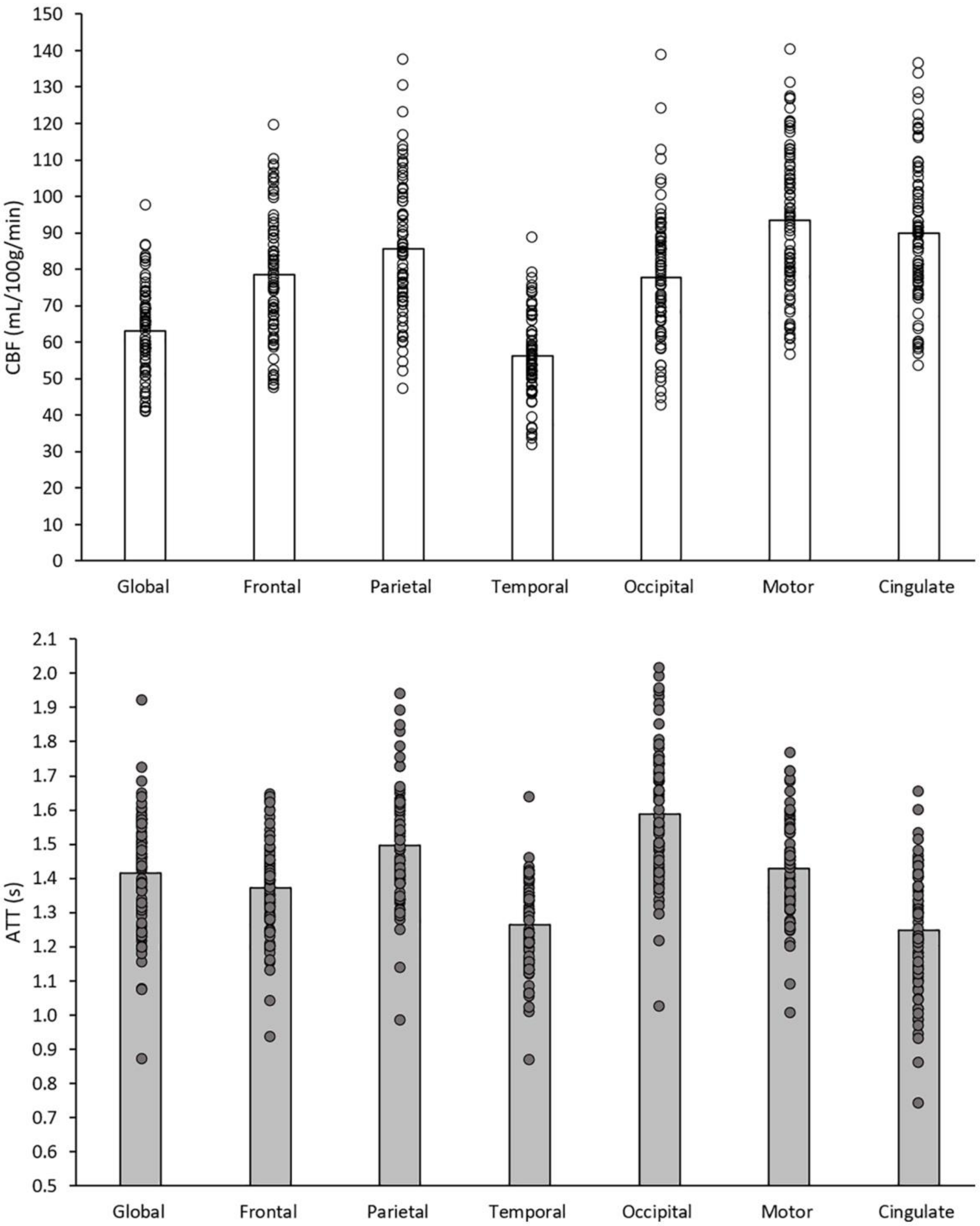

Supplementary Figure 1. Means and individual data for global and regional cerebral blood flow (CBF; top) and arterial transit time (ATT; bottom) in healthy older adults. Global (*n* = 78) and regional (*n* = 77) analyses were performed in native and MNI space, respectively.

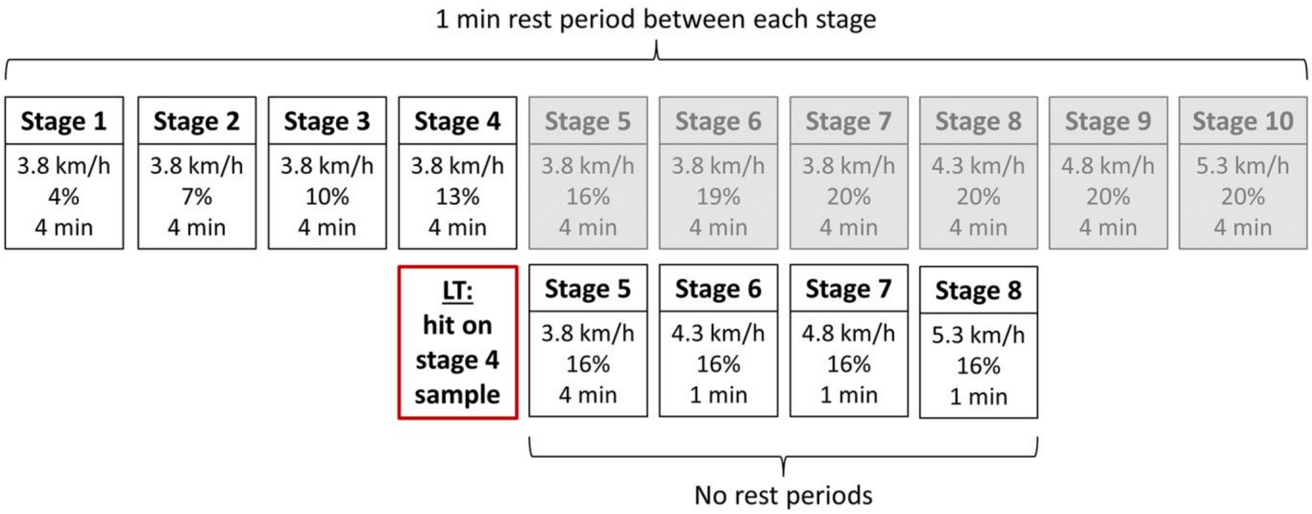

Supplementary Figure 2. Incremental treadmill test format example where lactate threshold (LT) is hit after stage 4. Stages in grey are possible stages had lactate threshold not been hit after stage 4.

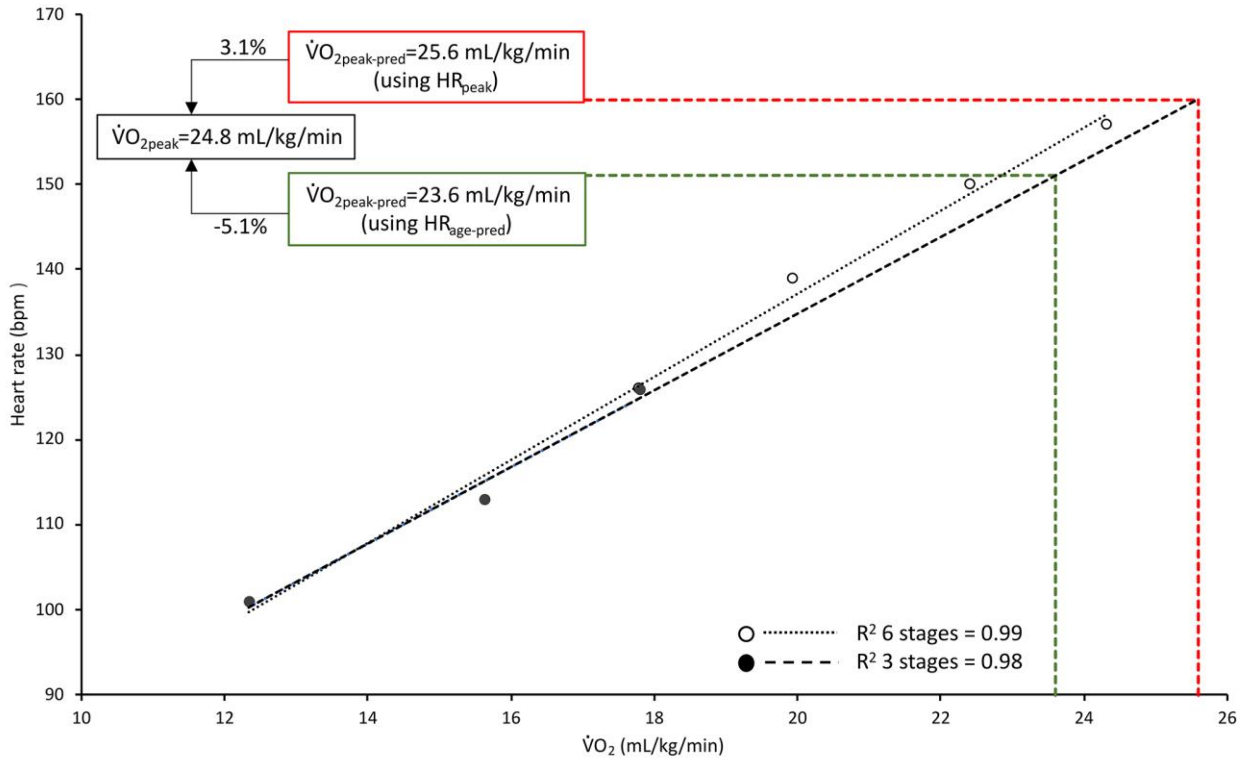

Supplementary Figure 3. Example of peak oxygen consumption ($\dot{V}O_{2peak}$) prediction from sub-maximal heart rate and $\dot{V}O_2$ data (*n* = 1).

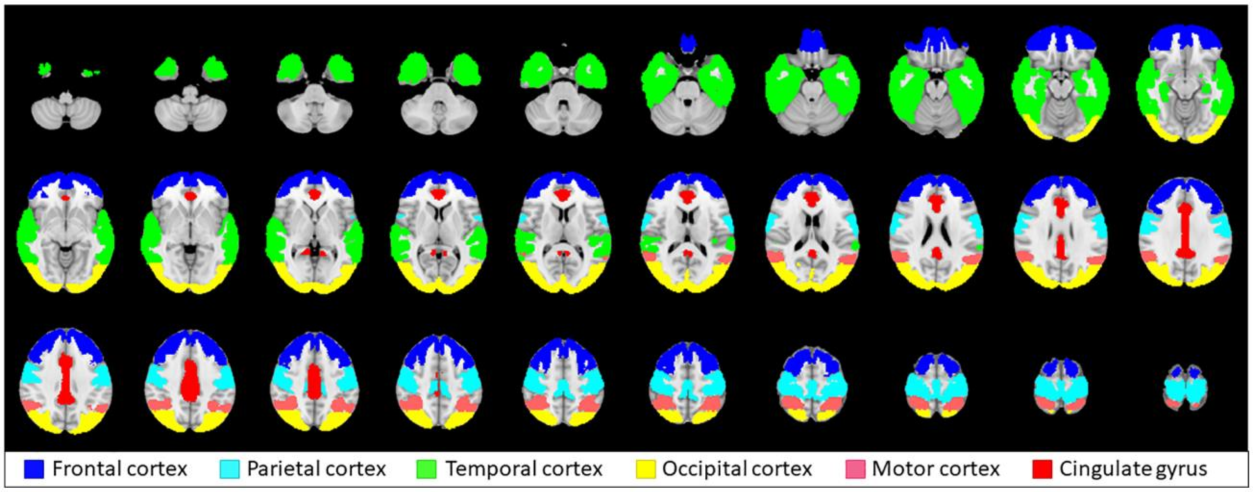

Supplementary Figure 4. Grey matter masks used for region of interest analysis in MNI space.

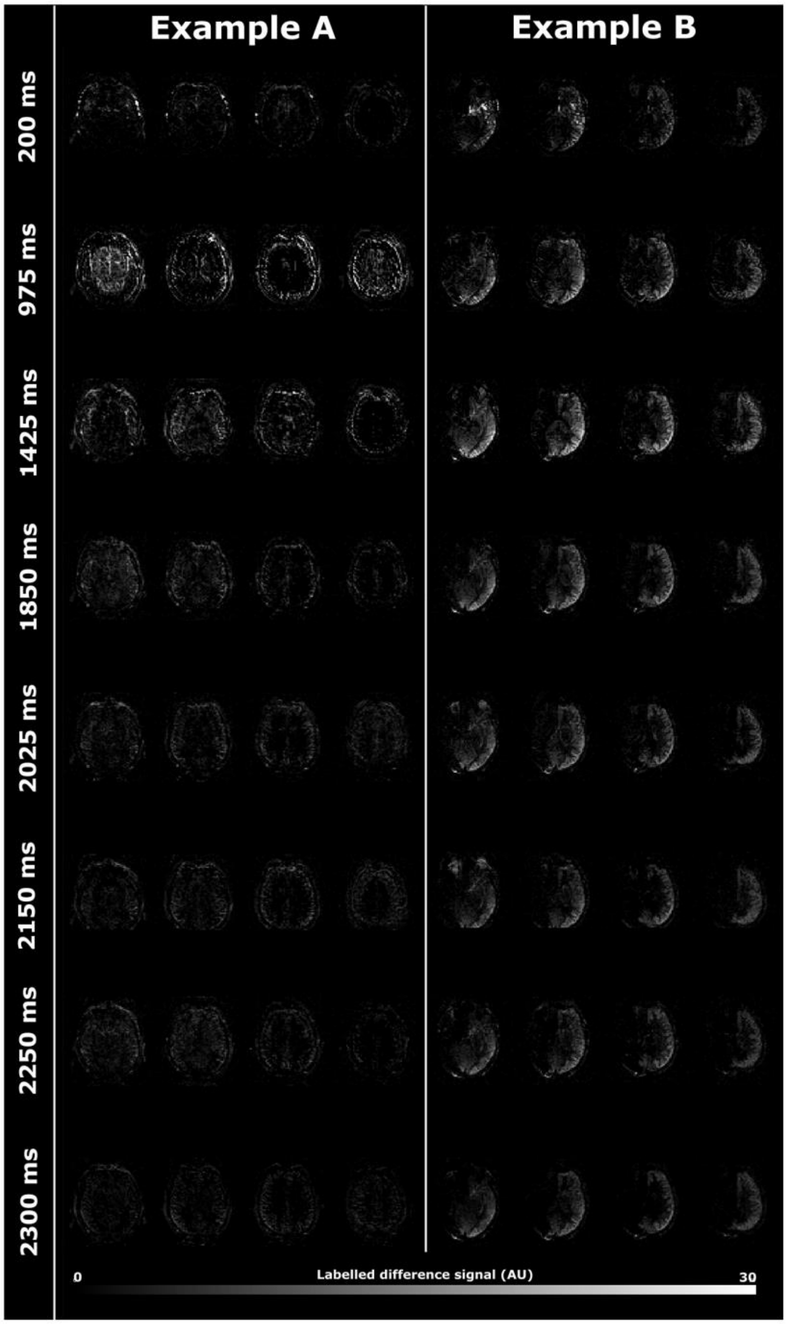

Supplementary Figure 5. Examples from two excluded participants of arterial spin labelling difference maps at each post-labelling delay. Abbreviation: AU: arbitrary units.
