# Supplementary material for: Determinants of cerebral blood flow and arterial transit time in healthy older adults
Source: Aging (Albany NY). 2024 Sep 18;16(18):12473–97. doi: 10.18632/aging.206112 (PMC11466485; doi:10.18632/aging.206112)
Supplement: Supplementary Tables [file aging-16-206112-s003.pdf]

SUPPLEMENTARY TABLES

Supplementary Table 1. Global and regional values for grey matter CBF and ATT.

	Total	Male	Female
**CBF (mL/100 g/min)**			
Global	63 ± 12	62 ± 12	65 ± 12
Frontal cortex	78 ± 17	78 ± 21	79 ± 18
Parietal cortex	85 ± 19	85 ± 23	86 ± 19
Temporal cortex	56 ± 12	55 ± 15	58 ± 11
Occipital cortex	78 ± 18	75 ± 21	81 ± 18
Motor cortex	93 ± 20	92 ± 25	95 ± 20
Cingulate gyrus	90 ± 20	87 ± 23	92 ± 20
**ATT (s)**			
Global	1.42 ± 0.17	1.43 ± 0.17	1.40 ± 0.17
Frontal cortex	1.37 ± 0.14	1.39 ± 0.26	1.35 ± 0.14
Parietal cortex	1.50 ± 0.16	1.54 ± 0.30	1.46 ± 0.15
Temporal cortex	1.26 ± 0.13	1.27 ± 0.24	1.26 ± 0.13
Occipital cortex	1.59 ± 0.19	1.63 ± 0.32	1.55 ± 0.19
Motor cortex	1.43 ± 0.14	1.45 ± 0.27	1.40 ± 0.14
Cingulate gyrus	1.25 ± 0.18	1.25 ± 0.26	1.25 ± 0.19

Values represent means ± standard deviation. Global (*n* = 78) and regional (*n* = 77) values are from native and MNI space, respectively. Abbreviations: CBF: cerebral blood flow; ATT: arterial transit time.

Supplementary Table 2. Associations between age, BMI, and cardiorespiratory fitness with regional CBF.

**CBF**	**Age**		**BMI**		**$\dot{V}O_{2peak}$**	
	**β**	***P***	**β**	***P***	**β**	***P***
Frontal	−0.07	0.613	−0.34	**0.008**	−0.16	0.346
Parietal	−0.05	0.722	−0.41	**0.002**	−0.13	0.446
Temporal	−0.12	0.361	−0.44	**<0.001**	−0.20	0.213
Occipital	−0.06	0.639	−0.43	**<0.001**	−0.08	0.597
Motor	−0.03	0.805	−0.35	**0.007**	−0.19	0.257
Cingulate	0.00	0.987	−0.39	**0.002**	−0.11	0.515

Separate multiple linear regressions were performed for each region (*n* = 77), independent variables: age, sex, BMI, and $\dot{V}O_{2peak}$. Bold indicates significant *P*-values survived adjustment for multiple comparisons. Abbreviations: β: standardised beta coefficient; CBF: cerebral blood flow; BMI: body mass index; $\dot{V}O_{2peak}$: peak oxygen consumption.

Supplementary Table 3. Associations between global CBF or ATT with cognitive function.

	gCBF (mL/100 g/min)		gATT (s)	
***n* = 76**	**β**	***P***	**β**	***P***
Age (years)	0.113	0.400	0.325	0.015
Sex	0.096	0.435	−0.107	0.372
Education	−0.079	0.522	0.075	0.532
Processing speed				
Response time	−0.049	0.696	−0.001	0.995
Accuracy	0.247	0.056	−0.082	0.514
Working memory				
2-back d prime	0.170	0.190	0.016	0.900
Attention (response time)				
Alerting	−0.122	0.344	0.041	0.746
Orienting	0.073	0.580	−0.017	0.896
Executive control	0.111	0.405	−0.034	0.795

Results from two multiple linear regression analyses. Abbreviations: β: standardised beta coefficient; gCBF: global cerebral blood flow; gATT: global arterial transit time.

Supplementary Table 4. Correlations between age and global cerebral blood flow (CBF).

Variables	Co-variates	Pearson correlation	*P*
Age/Global CBF		r (76) = −0.26	0.82
Age/Global CBF	Sex	r (76) = −0.32	0.78
